# Draft Genome Sequence of *Weissella confusa* MBF8-1, a Glucansucrase- and Bacteriocin-Producing Strain Isolated from a Homemade Soy Product

**DOI:** 10.1128/genomeA.01497-16

**Published:** 2017-01-26

**Authors:** Nicholas C. K. Heng, Chia-Wen Yeh, Amarila Malik

**Affiliations:** a Sir John Walsh Research Institute, Faculty of Dentistry, University of Otago, Dunedin, New Zealand; b Division of Pharmaceutical Microbiology and Biotechnology, Faculty of Pharmacy, Universitas Indonesia, Depok, Indonesia

## Abstract

We report here the draft genome sequence of *Weissella confusa* MBF8-1, an isolate from a homemade fermented soybean product that produces sucrases and exhibits antibacterial (bacteriocin) activity. The draft genome of *W. confusa* MBF8-1 comprises a 2.2-Mbp chromosome and a 17.8-kbp bacteriocin-encoding plasmid. Two putative glucansucrase genes were also identified.

## GENOME ANNOUNCEMENT

*Weissella confusa* (formerly *Lactobacillus confusus*) belongs to the *Lactobacillus*-*Leuconostoc* branch of the diverse Gram-positive lactic acid bacteria (LAB) group (1). Many LAB are important starter cultures in the fermentation of milk and food substrates and have long been a focus of attention for their production capabilities of either exopolysaccharides or antimicrobial peptides (1). *W. confusa* can be found in a variety of fermented food products and produces various types of novel exopolysaccharides that could be exploited for probiotic applications (1, 2). On the other hand, *W. confusa* is also regarded as an opportunistic pathogen and has been implicated in causing sepsis and bacteremias in humans (2).

*W. confusa* MBF8-1, a strain isolated from a homemade fermented soybean product in Tangerang, Indonesia, produces multiple exopolysaccharide (EPS) types (3). Homopolymeric EPS, such as glucans and fructans, are synthesized by glucosyltransferases (glucansucrases) and fructosyltransferases (fructansucrases), respectively (4). A previous investigation revealed that *W. confusa* MBF8-1 possesses at least two glucansucrase-encoding (*gtf*) genes in its genome (3). In addition to the production of EPS, *W. confusa* MBF8-1 produces weissellicin MBF, a narrow-spectrum bacteriocin (5, 6). Weissellicin MBF is plasmid encoded, and its biosynthetic locus resides on pWcMBF8-1 (17,643 bp), the first completely sequenced plasmid from *W. confusa* (6). These traits make MBF8-1 an attractive candidate for development as a probiotic strain and hence the rationale for sequencing its genome.

The *W. confusa* MBF8-1 genome was sequenced by a whole-genome shotgun strategy using a Roche GS-FLX+ pyrosequencer (1st BASE, Jakarta, Indonesia). A total of 60,537,716 bp (~28-fold coverage), generated from 149,271 quality-filtered sequence reads (average read length, 406 bp), was assembled using gsAssembler version 2.3 (Roche) and MIRA version 4.0 (7). The resulting contigs were initially annotated using the Rapid Annotations using Subsystems Technology (RAST) server (8), ordered with reference to the *W. confusa* LBAE C39‑2 and *Weissella ceti* WS105 genome sequences (9, 10), and finally annotated by the Prokaryote Genome Annotation Pipeline (PGAP [11]) for deposition in NCBI/GenBank.

The *W. confusa* MBF8-1 draft genome sequence totals 2,199,279 bp comprising 44 chromosomal contigs (2,181,636 bp, with an average G+C content of 44.7% and *N*_50_ value of 95,287 bp) and plasmid pWcMBF8-1 (17,643 bp). PGAP detected 2,088 coding sequences, five rRNA operons, and 81 tRNA genes in the MBF8-1 chromosome. Using the partial amino acid sequences of glucansucrases Gtf8-1A and Gtf8-1B (3), two complete open reading frames, *gtf8-1A* and *gtf8-1B*, respectively, were identified. A 1,769-amino-acid protein is encoded by *gtf8-1A*, and *gtf8-1B* encodes a 1,652-amino-acid protein. Both Gtf8‑1A and Gtf8-1B are predicted to be exported by the secretory (Sec)-dependent pathway (12) and are distinct from the *W. confusa* LBAE C39‑2 dextransucrase (13). Gtf8-1A is homologous to GtfKg3 of *Lactobacillus fermentum* Kg3 (14), and Gtf8-1B is more similar to GtfG of *Streptococcus gordonii* (15).

### Accession number(s).

This whole-genome shotgun project has been deposited in DDBJ/ENA/GenBank under the accession no. MNBZ00000000. The version described in this paper is the first version, MNBZ01000000. The GenBank accession number of plasmid pWcMBF8-1 is KR350502.
